# Actuator and Contact Force Modeling of an Active Soft Brace for Scoliosis

**DOI:** 10.3390/bioengineering9070303

**Published:** 2022-07-11

**Authors:** Athar Ali, Vigilio Fontanari, Werner Schmoelz, Marco Fontana

**Affiliations:** 1 Department of Industrial Engineering, University of Trento, 38123 Trento, Italy; vigilio.fontanari@unitn.it; 2 Department of Orthopedics and Traumatology, Medical University of Innsbruck, 6020 Innsbruck, Austria; werner.schmoelz@i-med.ac.at; 3 Institute of Mechanical Intelligence, Scuola Superiore Sant’Anna, 56010 Pisa, Italy

**Keywords:** soft brace, modeling, twisted string actuators (TSA), scoliosis, exoskeletons, wearable robotics

## Abstract

Scoliosis is an abnormality of the spinal curvature that severely affects the musculoskeletal, respiratory, and nervous systems. Conventionally, it is treated using rigid spinal braces. These braces are static, rigid, and passive in nature, and they (largely) limit the mobility of the spine, resulting in other spinal complexities. Moreover, these braces do not have precise control over how much force is being applied by them. Over-exertion of force may deteriorate the spinal condition. This article presents a novel active soft brace that allows mobility to the spine while applying controlled corrective forces that are regulated by varying the tensions in elastic bands using low-power light weight twisted string actuators (TSAs). This article focuses on the actuator and contact force modeling of the active soft brace (ASB). The actuator modeling is required to translate the twisting of string in terms of contraction of the string’s length, whereas the contact force modeling helps in estimating the net resultant force exerted by the band on the body using single point pressure/force sensors. The actuators (TSAs) are modeled as helix geometry and validated using a laser position sensor. The results showed that the model effectively tracked the position (contraction in length) with root mean square error (RMSE) of 1.7386 mm. The contact force is modeled using the belt and pulley contact model and validated by building a custom testbed. The actuator module is able to regulate the pressure in the range 0–6 Kpa, which is comparable to 0–8 Kpa pressure regulated in rigid braces. This makes it possible to verify and demonstrate the working principle of the proposed active soft brace.

## 1. Introduction

Scoliosis is a 3D deformity of the spine that severely affects the quality of daily living. In severe cases, scoliosis results in affecting the musculoskeletal, respiratory, and nervous systems. Over 600,000 people suffering from scoliosis are being treated every year [1]. Although the exact cause is often unknown, scoliosis is generally classified depending on its etiology: idiopathic, congenital, or neuromuscular [2]. Idiopathic scoliosis can further be subdivided according to the age of onset as infantile (age 0–3), juvenile (age 4–9), or adolescent (age 10 up to skeletal maturity) [3,4]. Congenital scoliosis is due to embryological malformation; thus, children are typically diagnosed at a very early age [3]. Neuromuscular scoliosis is associated with secondary factors such as spinal cord trauma, cerebral palsy, spina bifida, or muscular dystrophy and can occur later in life [5]. Among these three groups, idiopathic scoliosis tends to be the most prevalent worldwide [6] with approximately 2–4% of children between 10 and 16 years of age being diagnosed [4]. Initially, scoliosis is screened for via physical examination but only fully diagnosed by either CT scan, MRI, or X-ray [4]. Based on the degree angle, the severity of scoliosis is determined. Curves of 10 degrees or less are considered mild, between 10 and 50, moderate, while above 50 degrees is severe [6]. Curves under 20 degrees usually only require monitoring and thus no therapeutic intervention. Curves between 20 and 40° tend to require some form of bracing [4,7]. Severe scoliosis often requires surgery, typically spinal fusion [3]. Some risk factors for developing scoliosis include gender, age, ethnicity, and family history [6]. 

The general goal of bracing is to maintain the curve below 50 degrees upon patient maturation. Although effective, bracing tends to prevent curves from worsening rather than permanently correcting or improving [6]. The rate of surgery after bracing is between 11 and 42.5%, depending on the previous treatment methods employed [8]. If treatment was rather conservative, there is a greater chance of surgery [8]. Surgical options are considered when a curve exceeds 45 degrees in immature patients and 50 degrees in mature. The goal of surgery is to halt the progression and improve spinal curvature and balance [6].

The use of bracing was reduced in the early 20th century due to innovations in surgical treatment [9]. However, it again attracted attention when complications started to appear in surgical treatments in the late 20th century. Several rigid braces have been developed in the past with the aim of either correcting the Cobb angle or halting its progression. Milwaukee [10,11,12,13], Boston [14,15,16], Lyon [17,18], and Chêneau [19,20,21] braces are some of the often-applied rigid braces to treat scoliosis. These braces have different correction principles, and they are developed to treat different scoliosis curves. Braces require patients to wear them for 18 to 23 h per day to be most effective [22]. To increase patient compliance, nighttime bracing such as Charleston brace [23] and Providence brace [24] was introduced [23,24]. These part-time braces had an aggressive correction effect and were mostly used for single curve thoracolumbar scoliosis. A few soft braces such as SpineCor [25], SpinealiteTM [26,27], and TriaC [28], have also been developed in the past to enhance comfort and halt the Cobb angle progression.

Although rigid braces are considered to be more effective in the treatment of scoliosis [29]. There are certain shortcomings associated with the rigid braces: (i) rigid static nature of these braces largely limit mobility of the spine and can result in muscle atrophy, spine stiffness, and flat back issues; (ii) rigid braces affect cardiopulmonary efficiency; (iii) reduction in the physiotherapy exercise potential; (iv) long construction time; (v) socio-economic implications; (vi) rigid braces causes abnormal bone deformation and skin breakdown [9,30]. Soft braces on the other hand are more compliant and enhance comfort but have a less corrective effect. Soft braces can be used to halt the progression of the Cobb angle and, in some cases, correct it if the severity of the curve is not too high, and in specific growth phases [31]. Several clinical studies [32,33] have been carried out to compare the outcome of the soft and rigid braces. There is not enough evidence to deduce an explicit conclusion on the effectiveness of the interventions [26].

The underlying brace technology used in both rigid and soft braces has not significantly changed over the last 50 years and remains archaic. Both rigid and soft braces are passive and do not have precise control over how much force is being exerted by them over the spine. ROSE dynamic brace developed by Colombia University is the only active brace that uses two parallel Stewart platforms to exert forces on the spine [34]. ROSE was a big advancement in the field of an active corrective orthosis. However, the use of eight series elastic actuators significantly increases the power consumption and weight of the device. This limits the use of ROSE in clinical applications as the braces are supposed to be worn typically 18 h a day.

This article presents an active soft brace that allows mobility to the spine while applying the controlled corrective forces. The forces are being exerted by the elastic band in the form of elastic resistance whose tension will be controlled using twisted string actuators. This article mainly focuses on the modeling of the actuation mechanism and contact force between the brace and the body.

This article is organized as follows. Section 2 deals with the materials and methods. It also gives an overview of device working principle and design. Section 3 describes the results and is divided in two main sub sections: actuator modeling and contact force modeling. Section 4 includes a brief discussion on the results followed by the conclusions.

## 2. Materials and Methods

This article presents the actuator and contact force modeling of a newly designed active soft brace. The active soft brace exerts forces using elastic bands. Twisted string actuators are used to actively control the tension in the elastic bands to regulate the amount forces being exerted. The twisted string actuators are modeled as helix geometry. It is also important to find the relationship between the net resultant force applied by the elastic band on the torso and the localized contact force between the elastic band and the torso at a certain contact location. Therefore, the result section of this article is divided into two main sections. Modeling of the actuator and contact force modeling.

### 2.1. Device Working Principle and Design

**Figure 1:**
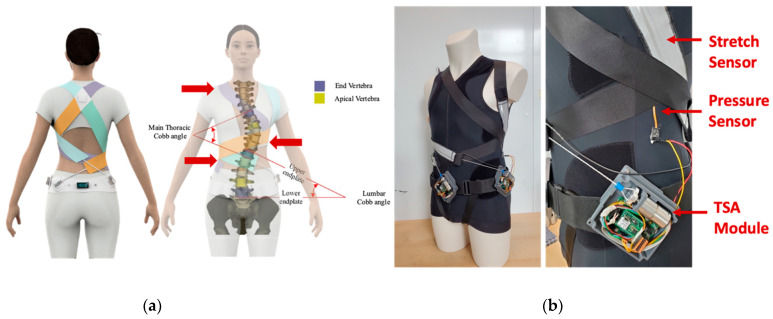
Active soft brace design and prototype. (**a**): conceptual design with scoliotic spine description; (**b**): prototype with actuator module, bands and stretch sensor.

The active soft brace uses four 50 mm wide elastic bands to apply the corrective forces to the spine. These bands are designed in such a way to provide thoracic rotation, shoulder rotation, and lateral bending (see Figure 1). The corrective elastic bands are attached to a contoured body vest. Firstly, the right thoracic flap (orange band) is attached to the lower right corner of the vest and wrapped around the rib cage to finally attach to the pelvic back of the body. This band provides thoracic rotation in a counterclockwise direction and is attached using Velcro crocodile strips. The tension in the band can be adjusted to keep the spine at the correct posture. The second flap (Tosca green band) is attached to the left thoracic base. This flap wraps around the abdominal part of the body and goes all the way to the right half of the pelvic back. Tension in this band is adjusted a bit less as compared to the orange flap to keep the spine rotated in a counterclockwise direction. The third band (purple flap) is attached to the left shoulder, rotates around the rib cage and back, and is finally attached in the front of the pelvic belt. This band generates clockwise shoulder rotation and left lateral flexion at T12. The fourth band, the right shoulder flap generates clockwise shoulder rotation and clockwise shoulder tilt. In the long term, this will reprogram the neuromuscular system and will be able to slow or stop the curve progression and improve the overall posture of the patient.

The elastic band revolves around the spine to apply corrective forces in the form of elastic resistance while allowing mobility to the spine. The amount of forces being applied by the brace is being controlled using twisted string actuators. Each elastic band has a stretch sensor attached to it to measure the stretch in the band and use that as feedback to design the control system. The design of the brace is inspired by the SpineCor brace. The goal is to provide 3D correction effect. The location of the bands can be adjusted with help of the doctors to patients’ specific curves to provide clockwise or anticlockwise rotation and managing the counterthrusts.

### 2.2. Actuation Module

**Figure 2:**
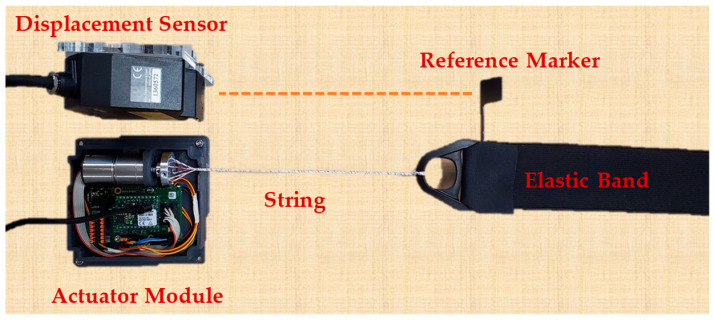
Test setup for actuator modeling (TSA actuation module with elastic band and laser displacement sensor).

The active soft brace actuation mechanism consists of Faulhaber^©^ Minimotor SA Drive Systems (Schönaich, Germany) DC motor (2214X006BXTH) with incremental encoder (IEF3-4096) and planetary gearhead (22GPT 4.5:1). Motion controller MC3001 P RS/CO is used to control the motor. The Dyneema fishing strings are attached to a mounting hub connected to the shaft of the motor to create a twisted string actuator. A twisted string actuator (TSA) is a simple, cheap, portable, and compact mechanism. In the TSA, a string that is co-axially attached to the motor shaft and converts rotational motion into linear displacement with a gear ratio that is quite high, which yields the potential to generate high output force with low input torque. When the motor twists the strings, they behave like a nonlinear transmission ratio as shown in Figure 2. A model of TSA was developed using helix’s geometry and details can be found in result section.

### 2.3. Contact Force Modeling

The active soft brace applies the force through elastic bands whose tension is being controlled using TSAs, modeled in the previous section. It is crucial to measure the force that the brace is exerting over the body. One critical point is that the contact force measured using a single point pressure/force sensor between the torso and the brace’s elastic band is not the net resultant force exerted by the elastic band over the torso. To realize this a testbed has been developed using a wooden dummy representing the thoracic side of the torso. The wooden dummy is connected to a load cell to measure the net resultant force and single-point pressure/force sensors to measure localized normal force at certain contact points. 

#### Testbed Setup

**Figure 3:**
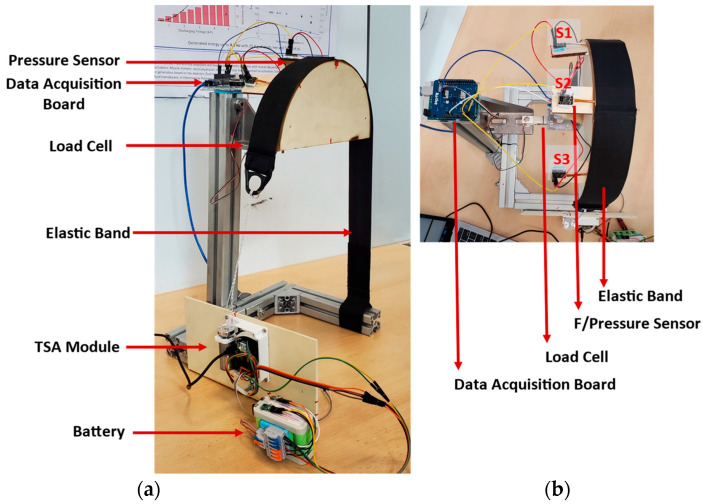
Testbed for contact force modeling. (**a**): Isometric view showing the actuator module, battery, bands and data acquisition board; (**b**): Top view with three single point pressure/force sensors.

A testbed has been developed to study the relationship between the force that we are measuring at a single point using three 4.5 N Singletact^©^ (Glasgow, UK) pressure/force sensors and a net resultant force applied by the elastic band on the torso using loadcell. The testbed configuration is shown in Figure 3.

Wooden blocks were cut into semi-circles of 250 mm diameter, using a laser cutter, and glued together to construct a shape 50 mm wide representing half torso. Duct tape was used to reduce the friction between the wooden surface and the elastic band. The wooden surface was fixed to a metallic structure through a load cell to measure the resultant force exerted by the elastic band. The force sensors were placed between the elastic band and the wooden surface at the angle of −45°, 90°, +45°. The sensors were protected using tape and a protecting sheet to avoid any sliding caused by the elastic band. The load cell and force sensors were calibrated incorporating the weight of the wooden block on the loadcell and the initial force of the elastic band over the force sensor. Section 3.2 describes the contact force modeling in detail.

## 3. Results

This section is divided into two main sections: (i) actuator modeling and validation; (ii) contact force modeling.

### 3.1. Actuator Modeling and Validation

**Figure 4:**
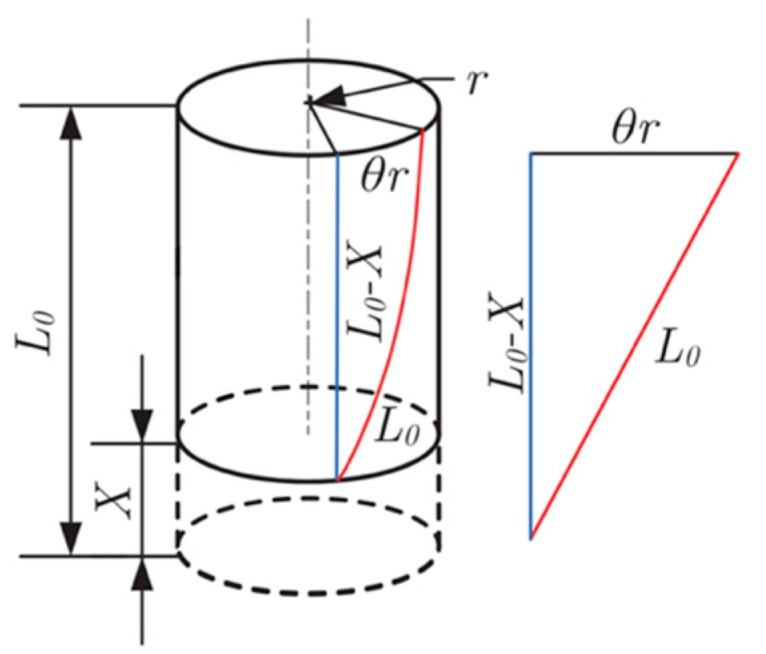
Helix’s geometry model for TSA.

To effectively control the twisted string actuation mechanism, it is important to estimate the contraction of the string length. This could be implemented with an external linear displacement sensor, but since we aim at realizing a low-cost/low-complexity system we aim at estimating the string length based on the rotation of the actuator shaft *θ* that can be measured through an encoder attached to the motor. The actual length of a twisted string can be derived from the string’s helix geometry as shown in the kinematic scheme presented in Figure 4 [35]. Using simple geometry considerations, the contraction of length X can be written as a function of twist angle *θ* can be written as:

(1) $$X=L_{0}-\sqrt{L_{0}^{2}-r^{2}\theta^{2}}.$$

where *L*_0_ is the length of the string bundle before twisting and r is the radius of the string bundle after 5 turns.

**Figure 5:**
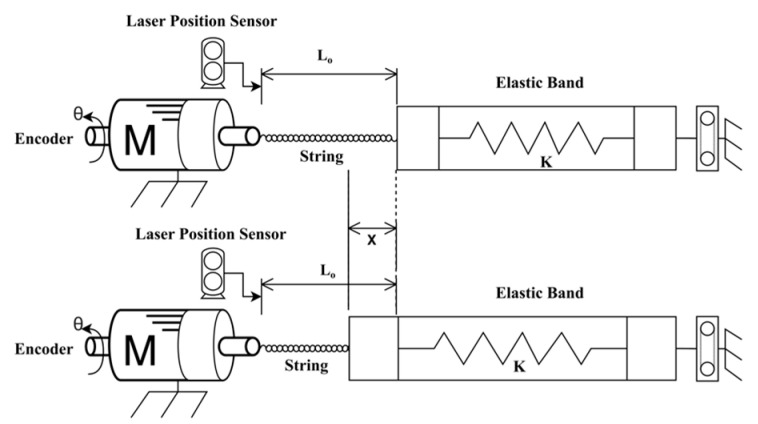
Actuator model validation setup schematic diagram.

To verify the actuation model of the active soft brace, a setup was designed consisting of Faulhaber^©^ MC3001 motion controller and DC motor module to twist the four Ø 0.4 mm Dyneema fishing strings of 20 cm (200 mm) attached to the motor shaft and elastic band. A laser displacement sensor (Keyence lk-g152, Mechelen, Belgium) was used to measure the actual position, as shown in Figure 4 and Figure 5.

**Figure 6:**
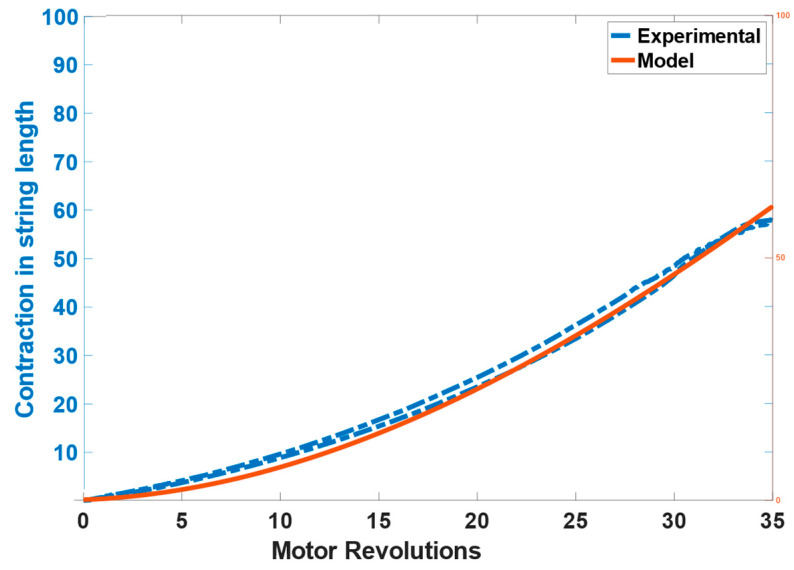
Actuator model validation results showing the contraction in string’s length.

The results of the contraction of the string length calculated through the model and measured from the laser displacement sensor can be seen in Figure 6. The model tracks the position effectively with the RMSE of 0.17386 cm (1.7386 mm).

The twisted string actuator uses strings to generate the pulling forces. There is a limit to how many rotations and cycles that strings can bear. Therefore, it is important to study the cycle of the twisted string actuator in different twisting regions. The cycle can be represented by twisting the actuator for a certain number of rotations, i.e., 20 30 35 35, and then returning to the original position. 

A test setup was developed to carry out a lifecycle test. It consists of a DC gear motor with incremental encoder, a mounting hub attached to the motor’s shaft, a string attached to the mounting hub and elastic band, and a motion controller. The motion controller was programmed to carry out cycles of the different number of turns (20 30 35 45). A few seconds delay was introduced after each cycle to keep the motor temperature lower. The motion controller can measure the current drawn by the motor and torque generated by the motor. The information related to the tests such as the number of turns (per cycle) and the current along with the time stamp was logged into the CSV file. This information is enough to determine the number of cycle strings endured by considering the failure point of the system through motor current.

**Figure 7:**
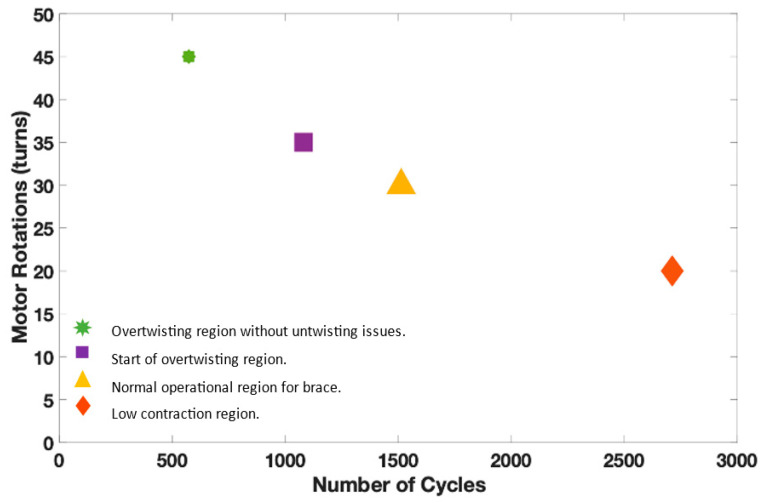
Lifecycle test of twisted strings with different twisting regions.

These numbers of turns were chosen based on the twisting regions, i.e., low contraction, over twisting, etc. The low contraction region which represents up to 20 turns showed a higher life cycle of 2712 compared to other regions. The maximum contraction limit without the overtwisting of the strings (35 turns) represents the start of the over twisting phase with the lifecycle of 1080. Overtwisting may cause untwisting issues, the limit where overtwisting is possible without untwisting issue is represented by 45 turns. Therefore, the active soft brace operates below the overtwisting region. Figure 7 demonstrates the life cycle of the TSA in different twisting regions. It can be observed that the life cycle reduces for higher contraction of the strings.

### 3.2. Contact Force Modeling

**Figure 8:**
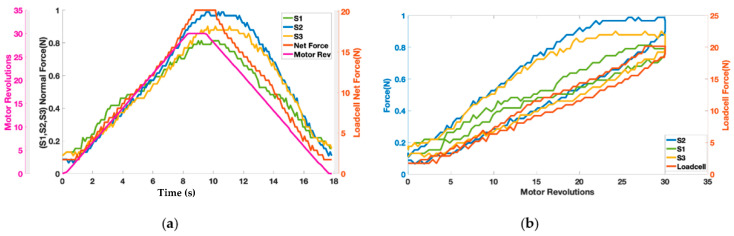
(**a**) Representation of contact reaction forces from single point force sensors and net resultant force along with (**b**) sensor hysteresis.

The motor twisted the string up to 30 revolutions resulting in pulling the elastic band and exerting the force on the wooden block shown in Figure 3. Figure 8a shows the change in force values of three Singletact pressure/force (S1, S2, S3) and loadcell force with respect to motor revolutions. The motor revolutions are displayed with a pink line with pink axis on the left while the forces of the single point pressure/force sensors are displayed with respect to black axis in green (S1) blue (S2) and yellow (S3). The value of the forces measured through the force/pressure sensors are quite low compared to the net normal force measured through loadcell (net force in red with right y-axis as reference). This shows trend/co-relation between the forces measured from the Singletact pressure/force sensors and net normal force measured through the loadcell. Sensors’ hysteresis are also plotted in Figure 8b.

It can be seen from Figure 8a that the net normal force that is acting on the torso (wooden dummy) is not equal to the force that is being measured with Singletact pressure/force sensors at single point. In a practical application, load cell cannot be placed on the body to measure net resultant force. Therefore, it is important to model that force.

**Figure 9:**
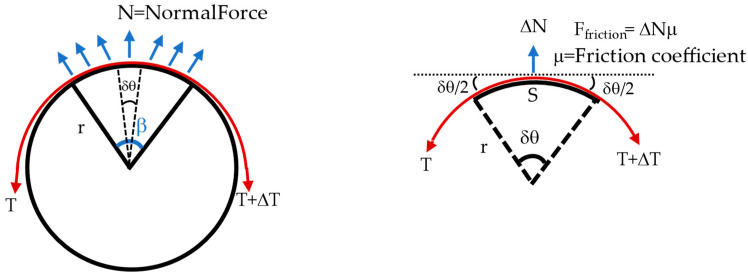
Belt pulley contact force model to model contact force where red represents the band.

The testbed can be modeled as a belt-pulley contact model (see Figure 9). Consider a belt wrapped over the pulley with a wrap angle β. The aim is to find the relation between the normal force measure at the segment of the wooden dummy (i.e., the force measured at point using single-point pressure/force sensors) with respect to net normal force that is acting on the wooden dummy (equivalent to the one measured by loadcell). Wrap angle β of the pulley can be divided into small segments of angle δθ. If the pulley is holding the belt and is in an equilibrium state, the sum of forces acting in x- and y-direction is equal to zero shown by Equations (1) and (2).

(2) $$\sum Fx=\left(T+\Delta T\right)\cos\left(\frac{\delta \theta }{2}\right)-T\cos\left(\frac{\delta \theta }{2}\right)-\Delta N\mu =0$$

(3) $$\sum Fy=\Delta N-T\sin\left(\frac{\delta \theta }{2}\right)-\left(T+\Delta T\right)\sin\left(\frac{\delta \theta }{2}\right)=0$$

From (2)

(4) $$\Delta T=\frac{\Delta N\mu }{\cos\left(\frac{\delta \theta }{2}\right)}$$

From (3)

$$\Delta N=2T\;\sin\left(\frac{\delta \theta }{2}\right)+\Delta T\;\sin\left(\frac{\delta \theta }{2}\right)$$

Solving for the above equation for the *T*.

$$T=\frac{2}{\delta \theta }\sin^{-1}\left(\frac{\Delta N-\Delta T\sin\left(\frac{\delta \theta }{2}\right)}{2}\right)$$

Inserting the value of Δ*T* from (4).

(5) $$\begin{array}{c} T=\frac{2}{\delta \theta }\sin^{-1}\left(\frac{\Delta N-\frac{\Delta N\mu }{\cos\left(\frac{\delta \theta }{2}\right)}\sin\left(\frac{\delta \theta }{2}\right)}{2}\right)\\ T=\frac{2}{\delta \theta }\sin^{-1}\left(\frac{\Delta N\left(1-\mu \tan\left(\frac{\delta \theta }{2}\right)\right)}{2}\right) \end{array}$$

From the general expression of belt and pulley system Equation (6) can be stated.

(6) $$\left(T+\Delta T\right)=Te^{\mu \beta }$$

The net normal force exerted on the pully (wooden dummy) would be equal in magnitude of the forces (*T* + (*T* + Δ*T*)) exerted on the both end of the belt.

*T* + (*T* + Δ*T*) = Net Normal Force (*N*) (7)

Equation (5) represents the relationship between the force measured through the single point pressure/force sensor at a small segment of angle *δθ* (Δ*N*) and the total normal force equivalent to what measured through loadcell. 

*N* = Net normal force (equivalent to loadcell value)

β = Wrap angle = π

*μ* = Friction coefficient = 0.1

F_Friction_ = Friction force

*r* = Radius of the pulley (wooden dummy) = 125 mm

*S* = Segment circumference = force sensor width = 15 mm

*δθ* = Angle of segment = 6.8796° = 0.1201 rad

Δ*N* = Normal force at force sensor location

**Figure 10:**
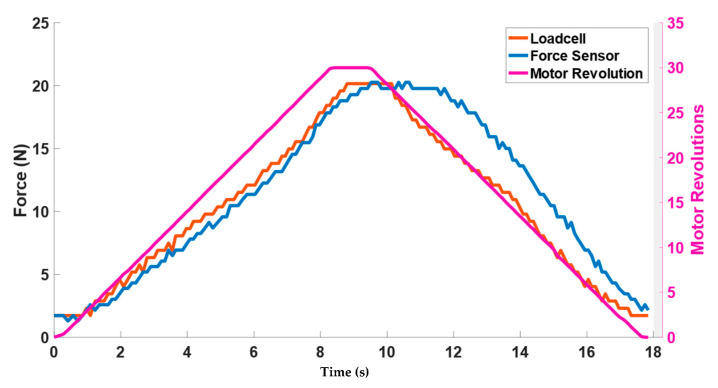
Contact force model validation results.

The results of the contact force model can be seen in Figure 10. Forces are plotted against the motor revolution (pink line) to show the change in the forces by using actuator (TSA). For simplification, only sensor S2, which is located in the middle, is plotted. The blue curve represents the modeled value of the force calculated from Equation (7). The blue curve closely tracks the net normal force value (red curve) measured through loadcell. It can be observed that during the untwisting phase, the modeled force value (blue curve) showed higher values than the loadcell curve. This is due to the sensor hysteresis reported earlier in the of Figure 8b.

Considering the contact forces that the active soft brace exerts on the wooden dummy one might wonder whether these force values are in line with the amount of pressure that the human body can comfortably sustain. Single-point pressure/force sensors were used to measure the pressure exerted by the elastic band. The Singletact 4.5 N pressure/force sensor used in the testbed has a diameter of 15 mm, resulting in area (A):

A = πr^2^ = 1.7671 × 10^−4^ m^2^

Pressure = Δ*N*/A

**Figure 11:**
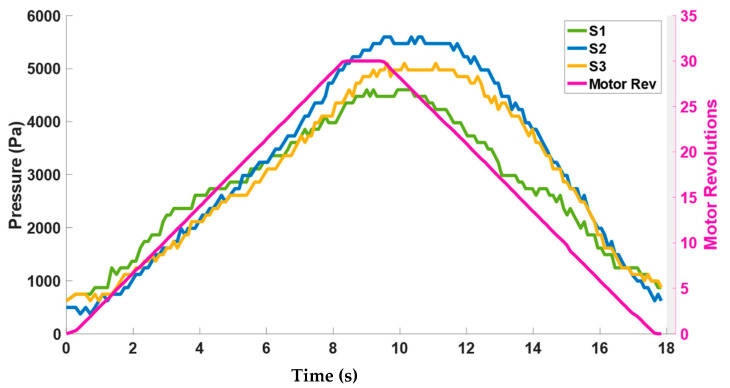
Corresponding pressure range exerted by the elastic bands.

Figure 11 plots the pressure calculated through the force values of the sensors S1–S3 with respect to actuator motor revolutions. It can be seen from Figure 11 that the amount of pressure that the band exerts is between the range of 0–6 KPa. These values of pressure are safe to be applied to the spine considering the pressure study performed by [36] in which a rigid brace exerted pressure through pads in the range of 0–8 KPa at different locations of the spine.

## 4. Discussion

The objective of the active soft brace is to apply controlled corrective forces to the spine while attempting to preserve physiological movement, which will help in reducing muscle atrophy, stop curve progression, enhance comfort and improve quality of life. 

The actuation model was developed using helix geometry and validated using a testbed with a Keyence lk-g152 laser position sensor. The model was simplified and followed the actual position quite well with RMSE 1.7386 mm. The TSAs significantly reduced the device weight and metabolic cost using low power 6 W DC motors. One TSA module weighs less than 200 g including motor (28.9 g), encoder, gear, motion controller, and mounting hub. In total the weight of the soft active brace is less than 2 Kg.

Modeling the contact force was one of the important aspects of this study. The contact forces measured between the elastic bands and the torso does not give information about how much net resultant force a band is applying on the torso. Another option could have been the use of pressure measurement systems (e.g., Tekscan^©^ pressure mats) to measure the distribution of the pressure around the torso under the influence of brace. These systems are quite expensive and have issues associated with following the geometry of the torso. Therefore, using single-point force/pressure sensors by PressureProfile^©^ and modeling the net resultant force was a more feasible solution. The pressure range exerted by the active soft brace is 0–6 Kpa and is comparable to the pressure reported for a rigid brace [36]. 

The brace actuation module is using a PID controller with position and force as feedback. The stretch sensors are being used as position feedback. We have studied the various fabric and silicon-based stretch sensors for measuring the device stretch; however, this was not in the scope of this article. 

This article focuses on the device working principle, actuation modeling, and contact force modeling. This article does not cover the clinical aspect of scoliosis treatment. A separate study has been conducted on a scoliotic spine finite element model and evaluation of in-brace correction. The finite element model in that work was validated with a vitro study and in-brace correction was observed by simulating different pulling forces through TSAs [37]. 

An active soft brace may have limited clinical applications. It may have potential usefulness only in specific subgroups of patients, due to weaker mechanical pushes provided, like other soft braces such as SpineCor brace. The device uses a twisted string actuator, which is light weight, low powered and compact, but the comfortability of the actuation module on the real subjects needs to be determined. The hysteresis of the twisted strings has not been considered. The active soft brace does not operate in overtwisted regions, but as the TSA goes into a higher twisted region, the hysteresis can affect the positional accuracy of the model.

The testbed used to validate the contact force model uses a wooden dummy. The testbed is rigid and encapsulated soft tissues are deformable. The effect of the skin/soft muscles might result in different contact forces. The single localization of study in the wooden test does not give sufficient information for completely understanding the correction effect. Often, more than one curve is present, and the indirect consequences of one-site correction are crucial. Therefore, future work includes the testing of the brace action on human subject. The first course of action would be to see in-brace correction of the patients while wearing a brace and studying the dynamic action of the active soft brace. This preliminary study helped in understanding whether the forces exerted by the brace are within the acceptable range and it gives useful insights into the mechanical working of the device.

## 5. Conclusions

In conclusion, the actuator and contact force models of an active soft brace have been developed and validated successfully. The actuator model developed through helix geometry showed a root mean square error of 1.7386 mm in position. The actuator model helped us to demonstrate the relationship between the motor rotation (*θ*) and stretch in the elastic band (contraction in string *X*). The contact force modeled showed how much amount force/pressure an active soft brace can regulate. Results showed that it can regulate between 0–6 kPa and is comparable to the pressure reported for the rigid braces 0–8 kPa. These results are quite useful in the development of the prototype and realizing the device’s mechanical working. 

## Data Availability

The data presented in this study are available on request from the corresponding author.
